# Hypermethylation of the *DLC1 *CpG island does not alter gene expression in canine lymphoma

**DOI:** 10.1186/1471-2156-10-73

**Published:** 2009-11-13

**Authors:** Jeffrey N Bryan, Mohamed Jabbes, Linda M Berent, Gerald L Arthur, Kristen H Taylor, Kerry C Rissetto, Carolyn J Henry, Farah Rahmatpanah, Wendi V Rankin, Jose A Villamil, Michael R Lewis, Charles W Caldwell

**Affiliations:** 1 Dept of Veterinary Medicine and Surgery, University of Missouri-Columbia, Columbia, MO, 65211, USA; 2 Department of Veterinary Clinical Sciences, Washington State University, Pullman, WA, 99163, USA; 3 Veterinary Medical Diagnostic Laboratory, University of Missouri-Columbia, Columbia, MO, 65211, USA; 4 Dept of Pathology and Anatomical Sciences, University of Missouri-Columbia, Columbia, MO, 65211, USA; 5 Dept of Internal Medicine, Division of Hematology/Oncology, University of Missouri-Columbia, Columbia, MO, 65211, USA; 6 Research Service, Harry S. Truman Memorial Veterans' Hospital, Columbia, MO, 65211, USA; 7 Ellis Fischel Cancer Center, University of Missouri-Columbia, Columbia, MO, 65211, USA

## Abstract

**Background:**

This study is a comparative epigenetic evaluation of the methylation status of the *DLC1 *tumor suppressor gene in naturally-occurring canine lymphoma. Canine non-Hodgkin's lymphoma (NHL) has been proposed to be a relevant preclinical model that occurs spontaneously and may share causative factors with human NHL due to a shared home environment. The canine *DLC1 *mRNA sequence was derived from normal tissue. Using lymphoid samples from 21 dogs with NHL and 7 normal dogs, the methylation status of the promoter CpG island of the gene was defined for each sample using combined bisulfite restriction analysis (COBRA), methylation-specific PCR (MSP), and bisulfite sequencing methods. Relative gene expression was determined using real-time PCR.

**Results:**

The mRNA sequence of canine *DLC1 *is highly similar to the human orthologue and contains all protein functional groups, with 97% or greater similarity in functional regions. Hypermethylation of the 5' and 3' flanking regions of the promoter was statistically significantly associated with the NHL phenotype, but was not associated with silencing of expression or differences in survival.

**Conclusion:**

The canine *DLC1 *is constructed highly similarly to the human gene, which has been shown to be an important tumor suppressor in many forms of cancer. As in human NHL, the promoter CpG island of *DLC1 *in canine NHL samples is abnormally hypermethylated, relative to normal lymphoid tissue. This study confirms that hypermethylation occurs in canine cancers, further supporting the use of companion dogs as comparative models of disease for evaluation of carcinogenesis, biomarker diagnosis, and therapy.

## Background

Dogs with spontaneously arising lymphoma represent a large animal model of naturally occurring non-Hodgkin's lymphoma (NHL) in a species which shares the human household environment and potential carcinogen exposure[1]. Lymphoma in dogs is common and shares similarities in cellular morphology and clinical behavior with the human disease [2-7]. The indolent forms of human NHL have a protracted course of disease that ultimately leads to therapy resistance and death[8,9] Lymphoma in dogs has a similar course of response to therapy followed by terminal resistance. As such, the dog has been proposed as a model for preclinical evaluation of novel diagnostics and therapeutics intended for human use[3,4]. To date, only p53, p16, and retinoblastoma tumor suppressor genes have been evaluated for mutation in canine NHL [10-12]. No published examination of possible hypermethylation of a tumor suppressor gene in a dog with NHL exists.

The *DLC1 *gene possesses tumor suppressor function[13,14]. The coded protein is a Rho-GTPase Activating Protein (RhoGAP) that counteracts the feed forward signaling of RhoA and Cdc42 among other Ras signaling proteins[15]. Loss of this function results in unconstrained growth signaling from the surface of the cell to the nucleus, changes in cell mobility through increased ROCK-mediated events, and signaling between the cell and its extracellular environment[13,15-17]. The tumor suppressor function has been confirmed by demonstrating that loss of *DLC1 *expression resulted in hepatocellular carcinoma formation in a knockdown mouse model[14]. Transfection of *DLC1 in vitro *caused decreased proliferation and colony forming potential of non-small cell lung carcinoma (NSCLC) cells and breast carcinoma cells [18-20]. Stable transfection of *DLC1 *in a mouse model of metastatic NSCLC halted tumorigenicity of the cell line and resulted in decreased invasiveness of the cells into normal tissue[18]. Expression microarray analysis of transfected cells revealed transcriptional upregulation of thrombospondin 2 (*TSP2*), a tumor growth inhibitor, acting principally through counteracting angiogenesis[19].

The human DLC1 protein contains three recognized functional domains: a sterile α motif (SAM), a RhoGAP, and a steroidogenic acute regulatory-related lipid transfer (START) domain domain[21]. The START and RhoGAP domains are necessary for the tumor-suppressor function of the protein[22]. Interaction with 14-3-3 protein binding in the linker area near the N-terminal SAM region appears to regulate DLC1 function[23]. At points of focal cellular adhesion, DLC1 interacts through a Src homology 2 (SH2) binding motif (Y422) with tensin family members cten and tensin2 at the SH2 domain on each protein[21,24]. This interaction was confirmed by mutation of the SH2 active regions of both *DLC1 *and *cten*, resulting in a loss of both interaction and localization at the plasma membrane[21]. DLC1 also interacts with tensin2 in caveolae, contributing to the organization of the local actin cytoskeleton and inhibition of the formation of stress fibers[24,25]. The interacting DLC1 and tensin2 suppress activity of the serum response element (SRE), a Ras cytoskeleton effector[25]. At focal adhesion sites, DLC1 function is modulated by binding of p120Ras-GAP[26]. Loss of such function could confer significant growth advantages to preneoplastic or neoplastic cells, contributing to the initiation, promotion, or progression of cancer, as well as metastasis. Indeed, *DLC1 *silencing has been demonstrated to be a significant contributor to many human cancers. This gene and its protein have not yet been characterized in the dog.

The presence of a hypermethylated promoter region of the *DLC1 *gene has been demonstrated in humans with NHL[17,27]. Shi and colleagues examined NHL cell lines and patient samples for hypermethylation of CpG islands with differential methylation hybridization (DMH) using a CpG island microarray[27]. The *DLC1 *gene was found to be hypermethylated in all six NHL cell lines examined, with concomitant silencing of transcription. In several lines, expression could be upregulated by treatment with a combination of a demethylating agent and a histone deacetylase inhibitor. Seventy-five NHL patient samples were examined for hypermethylation of several candidate genes, including *DLC1*. Of these, 87% demonstrated hypermethylation of *DLC1*[27]. Overall, expression of mRNA for this gene was significantly downregulated in tumor tissue compared to normal tissue[27]. Only recently have epigenetic mechanisms begun to be examined in dogs[28]. Initial work by our group has identified strong evidence that the tumor suppressor gene *DLC1 *is frequently hypermethylated in canine NHL, as it is in human NHL[27,29].

The purpose of this series of experiments was to sequence the canine *DLC1 *gene and determine whether hypermethylation of the gene is present in canine lymphoma. No literature or databank information characterizes this gene in dogs. While new, more precise prediction software has identified a putative structure for the canine *DLC1 *gene, no biological data confirms its existence or its expression in canine tissue. The studies presented here define the sequence of the mRNA, characterize the similarities and differences of the structure of the dog and human promoter regions of *DLC1*, report expression levels in the normal lymphoid samples and canine NHL samples, and identify DNA methylation patterns and their relationship to the expression of the gene.

## Results

**Figure 1:**
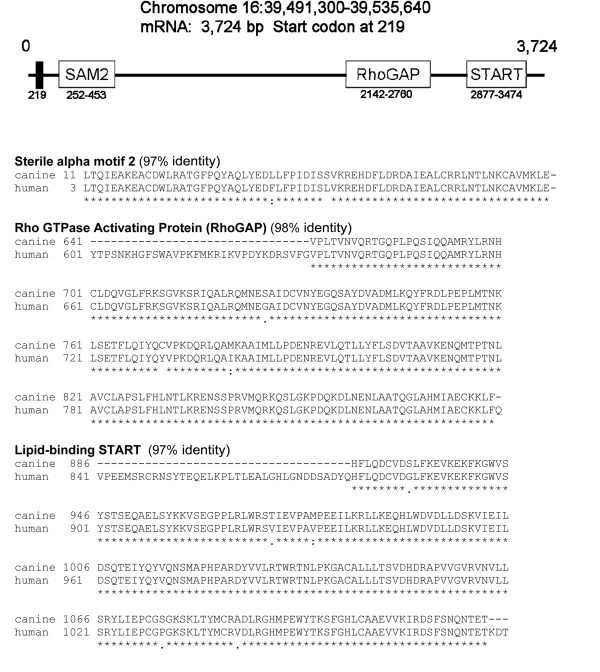
**Structure of the canine *DLC1 *gene based on sequencing results from mRNA isolated from normal spleen tissue**. The results have been deposited in GenBank with the accession number NM_001145071.1. The relative location of the protein translation start codon and functional groups in the mRNA are diagrammed, including sterile alpha motif 2 (SAM2), Rho GTPase Activating Protein motif (RhoGAP), and the lipid START motif. The location of each is described by the base number of the mRNA. The amino acid sequences for each functional group are depicted in parallel with the human sequence. The location of each peptide sequence is described by the residue number. The protein is 1083 amino acids long.

The construction of the canine *DLC1 *cDNA (GenBank FJ602870) as determine by 5'RACE is presented in Figure 1. It is constructed highly similarly to the human *DLC1*, (GenBank AF026219)[13]. The canine cDNA sequence shares 88% identity with the human sequence. The peptide sequences share 89% identity. All major human exons are represented in the canine sequence. The SAM, RhoGAP, and START domains are also present in the canine sequence with extremely high homology of 97% or higher. The SH2 interacting domain (hs Y442; cf Y450) and surrounding sequence is preserved in the dog (data not shown). The arginine finger (hs R677; cf R684) of the RhoGAP is also preserved in the dog (Figure 1).

Conserved motif analysis of the promoter sequences revealed strong homology between dog and human. There is a particularly strong conservation of Sp1 binding motifs. Of the canine Sp1 sites, 69% are common to human and 68% of the human sites are common to dogs. Furthermore, within the specific forms of the sites, interaction with pathway signaling is largely common between the two, including *jun*, dihydrofolate reductase, and gamma-globulin signaling pathways. Differences observed include cyclooxygenase signaling Sp1 in dogs and heat shock protein 70 signaling Sp1 in humans. Relative to human, the canine sequence has a higher density of CpG dinucleotides. The dog Sp1 sites are also more densely packed at the 5' end of the promoter region relative to the human sequence.

The conservation of non-Sp1 transcription factor binding sites is less striking. Of the conserved elements, only 41% are common to both the human and canine sequences. Conserved elements common to both species include ras and jun pathway binding sites.

Patient demographic data is presented in Table 1. The mean and median ages of the dogs with NHL were 7.8 and 7 years, respectively (range: 5-13 y). The mean and median weights of the dogs were 24.6 kg and 22.7 kg, respectively (range: 5.6-48.4 kg). Nineteen dogs were diagnosed with B-cell lymphoma and 2 with T-cell lymphoma.

**Table 1:** Demographic data of dogs in the study

Patient #	Sex	Breed	Age	BW	B/T	Stage	Survival	Diagnosis
**1**	FS	Am Co Sp	13	14.5	B	IV	706	Lymphoma
**2**	FS	Affenpinscher	12	5.6	T	IV	14	DSLL
**3**	MC	Mix	5	11.7	B	IV	52*	DLCL
**4**	FS	Mix	11	30.4	B	IV	104	DLCL
**5**	MC	Engl Spring	6	22.7	B	III	605	DLCL
**6**	MC	Mix	8	22.7	B	IV	238	DLCL
**7**	FS	Mix	7	22.5	B	IV	458	LBL
**8**	MC	Lab	6	43.7	B	III	448	DLCL
**9**	MC	Staff Terr	10	34.8	B	IV	6	FL II
**10**	MC	Mix	12	28.1	B	IV	754@	DLCL
**11**	FS	Schnauzer	11	10	B	N/A	231	Lymphoma
**12**	FS	Welsh Corgi	7	17.7	B	V	972@	DLCL
**13**	MC	Blue Tick	7	34.1	B	III	266	LBL
**14**	MC	Collie	5	32.2	T	II	97	Lymphoma
**15**	FS	Engl Spring	5	25.7	B	V	352	FL III
**16**	MC	Rottweiler	5	36	B	V	684	Burkitt
**17**	MC	Am Co Sp	8	15.2	B	IV	324	Lymphoma
**18**	MC	Golden	5	48.4	B	III	128	DLCL
**19**	FS	Beagle	9	11.6	B	V	140	DLCL
**20**	FS	Mix	5	17.5	B	V	427	DLCL
**21**	FS	Golden	5	34.2	B	IV	270	Lymphoma

Abbreviated breeds include American cocker spaniel (Am Co Sp), English springer spaniel (Engl Spring), Staffordshire terrier (Staff Terr), Blue tick hound (Blue Tick), Golden retriever (Golden) and mixed-breed dogs (Mix). Age is listed in years. Body weight is listed in kilograms. The immunophenotype (B/T) is listed. The stage is according to the World Health Organization staging standards. Survival is listed in days. Survival times marked with "@" to denote dogs alive at final analysis or lost to follow-up. Diagnosis is listed according to the Working Formulation where the tissue blocks could be retrieved or as lymphoma where they could not. Abbreviations for the diagnoses are as follows: DSLL, diffuse small lymphocytic lymphoma; DLCL, diffuse large cell lymphoma; LBL, lymphoblastic lymphoma; FL II, follicular lymphoma II; FL III, follicular lymphoma III.

**Figure 2:**
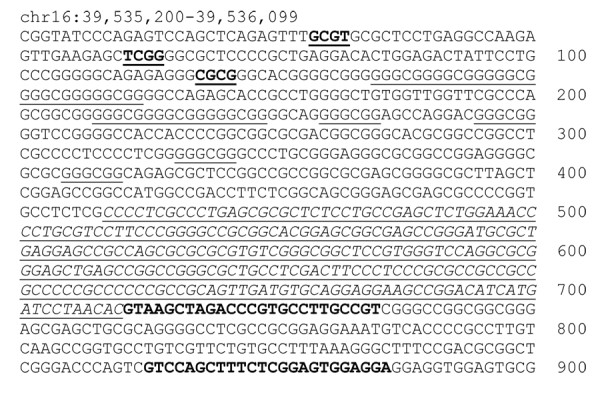
**CpG island and promoter regions of canine *DLC1 *with methylation assay sites identified**. The amplicon for the COBRA analyses spans from nucleotide 4 through 190. Restriction enzyme cut sites are marked in bold and underlined in this section. The Sp1 binding sites are in plain text and underlined. The BLAST comparison with the human promoter identified the region from nucleotide 100 to 675. The Promoterscan prediction identified the region that is italicized and underlined as likely to be the promoter, the final 38 nucleotides of which are within the first exon. The MSP primer sites are bolded at the 3' end of the sequence. 5' RACE determined the transcription start site to be at base 466, Chromosome 16:39,535,666.

**Figure 3:**
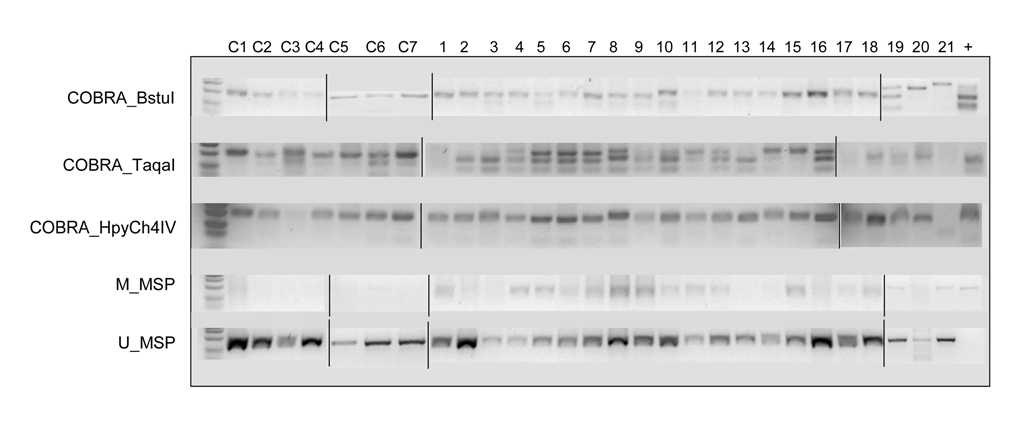
**Composite representation of original gels for COBRA and MSP results**. Control animals are listed C1-C7. Cases are listed 1-21. Positive methylation controls are in the far right lane. Molecular makers are in the far left lane. Depicted are the COBRA results with digestion using BstuI, TaqaI, and HpyCh4IV. Methylation specific PCR results are depicted in the fourth row for the methylated primers and the fifth row for the unmethylated primers. All faint bands in the initial gels were confirmed in repeated assays.

The results of the combined bisulfite restriction analysis (COBRA) are presented in Table 2. The BstuI cut-site, located centrally in the amplicon (Figure 2) was methylated in seven of 21 cases, with no methylation detected by COBRA in the seven normal samples (Figure 3). Methylation of this site was not significantly associated with the lymphoma phenotype (P = 0.141). The TaqaI cut-site demonstrated methylation in 19 of 21 cases, frequently complete in its cutting. This site was also weakly positive in two of seven normal samples (Figure 3). Methylation at the TaqaI site was significantly associated with the lymphoma phenotype (P = 0.009). The HpyCh4IV cut-site showed visual evidence of methylation in every case and four of seven normal samples (Figure 3). By gel software analysis, however, 11 of 21 cases and three of seven normals were deemed positive. Methylation of this site was not significantly associated with the lymphoma phenotype (P = 1.00). At the 3' end of the CpG island, 17 of 21 case and no normal samples were positive for methylation by methylation-specific PCR (MSP) (Figure 3). Methylation at this site was significantly associated with the lymphoma phenotype (P < 0.001). The relative ratios of the methylated bands and total lane intensity are presented in Table 2. Hypermethylation was not significantly associated with lymphoma classification or survival.

**Table 2:** Results of COBRA and MSP methylation analysis and real time expression analysis

Dog	B or T Cell	Rel Intensity Meth at HpyCh4IV	Rel Intensity Meth at TaqA	Rel Intensity Meth at BstuI	Meth:Unmeth Ratio for MSP	Relative Expression
**C1**	Normal	0.0461	0	0	0	0.3618
**C2**	Normal	0	0	0	0	N/A
**C3**	Normal	0	0	0	0	0.0103
**C4**	Normal	0	0	0	0	N/A
**C5**	Normal	0	0	0	0	N/A
**C6**	Normal	0.142	0.1148	0	0	0.0175
**C7**	Normal	0.1234	0.0573	0	0	0.0248
**1**	B	0	1	0	0.2666	0.0686
**2**	T	0.1961	1	0	0.0661	0.0992
**3**	B	0	1	0.2625	0	0.0752
**4**	B	0.1501	0.7505	0	0.9251	0.0164
**5**	B	0.1616	0.6651	0.4209	0.4932	0.1984
**6**	B	0.1821	0.7968	0	0.2628	0.0034
**7**	B	0.1407	0.1093	0.1042	0.3039	0.0260
**8**	B	0.1345	0.4357	0.2742	0.3431	0.2078
**9**	B	0	1	0.2481	0.4366	0.1575
**10**	B	0.1794	1	0.3558	0.1679	0.0655
**11**	B	0	0.2191	0	0.5008	0.2127
**12**	B	0	1	0	0.17993	0.0412
**13**	B	0	1	0	0	0.0884
**14**	T	0.1428	0.0655	0	0	0.0508
**15**	B	0	0	0	0.3781	0.0171
**16**	B	0.0351	0.4653	0	0.0614	0.1063
**17**	B	0	1	0	0.1700	3.0314
**18**	B	0.116	1	0	0.1543	0.1895
**19**	B	0.1654	1	0.4372	0.1366	0.2806
**20**	B	0	1	0	0	0.1403
**21**	B	0	0	0	0.1355	0.0000
Totals		11/21	19/21	7/21	17/21	
P value for lymphoma		1.00	0.009	0.141	<0.001	

There are seven control and 21 lymphoma samples. COBRA results are described as relative intensity of the methylated band out of all bands in the lane. The MSP results are described as a ratio between the methylated and unmethylated primer sets. Because these primer sets use different conditions for amplification, the ratio cannot be directly interpreted as a proportion of methylated DNA, but as a number relative to the other samples. Samples marked N/A were unavailable for the particular analysis.

**Figure 4:**
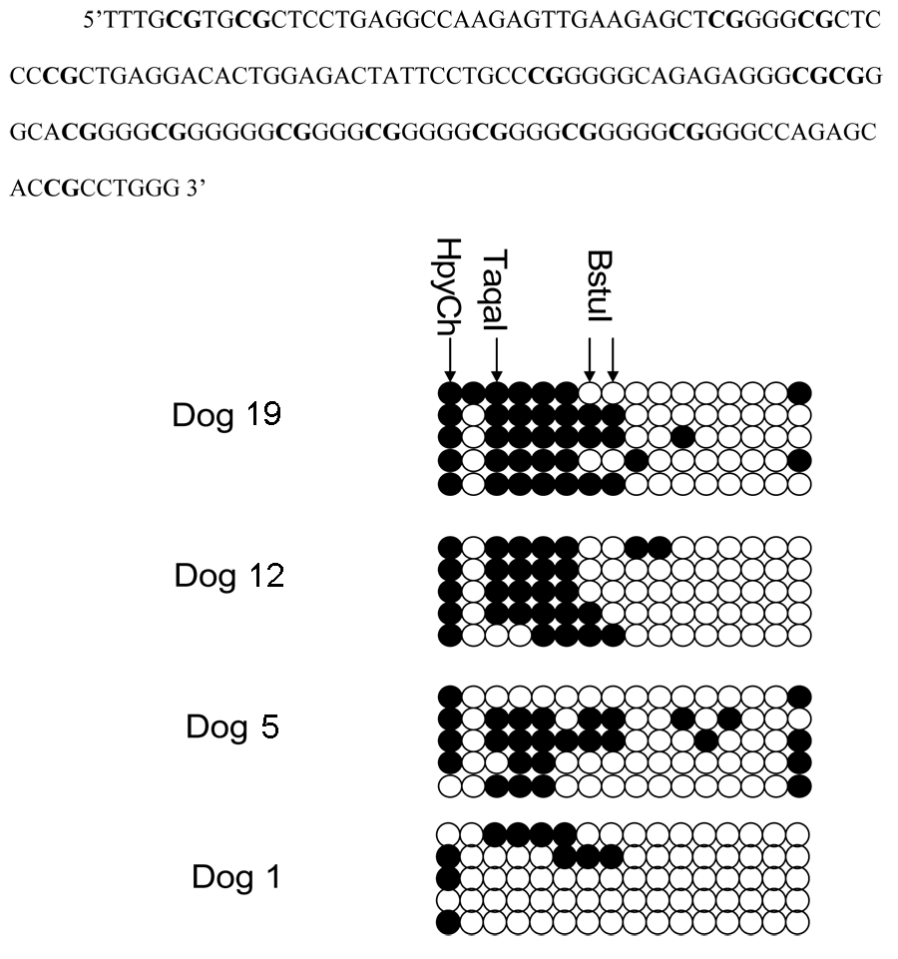
**Bisulfite sequencing results for normal dog 1, and dogs 5, 12, and 19 with NHL**. The corresponding DNA sequence is above with each CpG dinucleotide marked in bold. Open circles denote unmethylated CpG dinucleotides and black are methylated. Five clones for each dog were submitted for sequencing. Note the relatively lower methylation density for the normal dog. The high frequency of methylation at the HpyCh4IV cut-site in all dogs, including the normal dog, suggests that this CpG behaves as a genomic CpG dinucleotide and is routinely methylated. Note the minimal methylation in the right half each set of clones where the Sp1 sites reside.

Bisulfite sequencing data is presented in Figure 4. Five clones each from control dog 1 and lymphoma dogs 5, 12, and 19 were sequenced. As predicted by the pattern of COBRA results, methylation was present in the 5' region of the sequence at a visibly greater density than at the 3' of the amplicon. Density of methylation was the least in the normal Dog 1, with sporadic methylation more prominent at the 5' end near the edge of the CpG island. Density was significantly different from normal for lymphoma Dog 19 (P = 0.032). Dog 5 displayed slightly less methylation at the TaqaI cut-site than either dog 12 or 19. Dog 12 displayed the least methylation at the BstuI cut-site, with dog 5 intermediate between 12 and 19. All dogs displayed near complete methylation of the HpyCh4IV cut-site as suggested by the visual analysis of the gel, although methylation was slightly less complete in the normal dog. The small, diffuse nature of the band prevented adequate digital analysis in 10 of the cases. The AP-2 transcription factor binding site is located at the sixth CpG dinucleotide, and the ninth through fifteenth are included in Sp1 binding sites.

The relationship between age and relative methylation at each of the assay sites was examined using linear regression analysis. The relationship identified at the TaqaI site was positive (r = 0.394), but not significant (P = 0.086). The calculated power of the analysis was also low (0.403). This was the highest correlation identified. Normal samples were not included in this analysis, as they belong to a separate class, and the effect of lymphoma could not be separated from the effect of age.

Expression was variable in both normal and lymphoma samples, ranging from 0 to 3.03 relative to the GAPDH expression within the matching sample. Only for dog 21 was the expression undetectable, and this dog demonstrated methylation only in the MSP assay. There was no statistically significant relationship between methylation at any site and the expression of the gene in these NHL samples (P = 0.423).

## Discussion

Inactivation of the tumor suppressor gene *DLC1 *by deletion has recently been shown to occur nearly as frequently as p53 deletions in common human cancers[14]. Epigenetic silencing of the gene has been identified in cell lines and primary tumors of non-small cell lung, neuroectodermal, breast, colon, prostate, gastric, renal, uterine, esophageal, naspharyngeal, and rectal cancers, as well as NHL[15,18,30-35]. To better characterize lymphoma in dogs as a model for the human disease, we sequenced the canine *DLC1 *gene and evaluated the effect of hypermethylation on its expression. Sequencing of the 5' region of the cDNA was made difficult by the high GC content. The sequence presented here was isolated from normal canine spleen, and is highly similar to that reported by Yuan and others, isolated from human liver tissue[13].

Both human and canine sequences originate immediately downstream from a large CpG island, contain the same number of exons, and share protein functional groups. A gap is present in the canine genome build 2.0 in chromosome 16 from 39,504,330-39,505,008. A portion of the fifth exon of the *DLC1 *sequence lies within this gap, further revealing the sequence of the canine genome. The similarities in the canine and human amino acid sequences and in the transcription factors capable of binding to the control regions of these orthologs suggest highly common functions between the two. The promoter analysis presented here is a significant expansion from that previously reported[29]. Although specific pathway interactions are impossible to predict simply by the presence of conserved elements, the similarities conserved, and the differences that have arisen, are striking between the canine and human promoter elements of *DLC1*. The conservation of Sp1 binding sites between dog and human is high. The canine Sp1 sites are collected slightly more 5' and more densely relative to the human, with a less dense distribution of sites throughout the first exon and first intron region, which may have functional impact on methylation of the region. These findings suggest significant commonality of transcription control between dogs and humans, with subtle alterations introduced during the years of divergent evolution.

The extremely high GC content of this CpG island limited bisulfite evaluation to the margins of the island. The COBRA and MSP results demonstrate a pattern of greater density of methylation in the 5' and 3' flanking regions of the promoter of the canine gene with lower density of methylation near the core of the promoter region where, presumably, transcription is controlled. The greater number and density of Sp1 binding sites, relative to the human gene, may provide a boundary region, preventing the spread of methylation into the core of the promoter, even in neoplastic lymphocytes[36]. The HpyCh4IV cut site, the first CpG in the bisulfite sequencing analysis, is also the first CpG dinucleotide included in the island in the Methprimer analysis. This CpG dinucleotide may function as a genomic CpG, rather than as part of the island, and be methylated in almost every animal. The high rate of methylation in the bisulfite sequencing analysis supports this, as does the visual analysis of the gel image. Only the TaqaI site and the MSP data were significantly correlated with the neoplastic phenotype. Of these two, MSP is likely the better discriminator because methylation was present only in the NHL samples. Methylation at the BstuI site should continue to be evaluated in larger trials, as no normal sample was hypermethylated at that location on COBRA analysis.

The bisulfite sequencing results provide a visual representation of the likely control apparatus of this canine gene. AP-2 does not appear to contribute significantly to the expression of canine *DLC1*, as the binding site was uniformly methylated in two of the three lymphoma samples and partially in the normal sample. It is possible that the high density of Sp1, or another less frequent transcription factor, overwhelms any contribution of AP-2, as well protects other transcription factors within the core of the promoter region from hypermethylation. There is methylation in only a small minority of the Sp1 binding sites. This is in contrast to the methylation pattern identified in *DLC1 *in human prostate cancer in which the Sp1 sites are more heavily methylated, contributing to silencing[37]. An Sp1 boundary phenomenon has been reported in the *BRCA1 *promoter of breast cancer cells[38]. *In vitro *the Sp1 binding sites were shown to serve as normal boundaries between the hypermethylated and hypomethylated regions at the border of the gene control region[38]. Furthermore, methylation of these binding sites resulted in inhibition of binding by Sp1. This boundary effect was abrogated by specific mutation in a mouse model examining the *Aprt *gene[36]. In fact, no relationship appears to exist in the samples studied between the methylation status of the examined CpG island regions and the expression of this gene. Expression was present in all but one sample, and was highest in one of the lymphoma samples. This is not surprising, given the apparent protection of the majority of the core promoter binding sites identified. Discordant methylation and expression has been reported in the human gene *hTERT *by several investigators [39-41]. A critical, hypomethylated region has been discovered around the transcription start site (TSS) of the *hTERT *gene that allows transcription in spite of heavy methylation upstream[42]. It is likely that the canine *DLC1 *gene is similar, in that methylation not present in normal tissue can be readily demonstrated in the CpG island of canine NHL samples, but the core of the promoter appears unmethylated, allowing transcription to occur. Overall, expression of the gene is low in both node tissue and circulating lymphocytes, as found in humans by Shi and others[27]. The highest mRNA expression in this series was in lymphoma dog 17, but protein expression was not evaluated.

Of the 21 cases of lymphoma in this study, two were of T-cell immunophenotype. Unlike in our previously reported study, hypermethylation was identified in both cases. Methylation has been reported in T-cell lymphoid neoplasia. DLC1 was hypermethylated in three of six human patients with T-cell ALL[43].

Genomic hypomethylation and accumulation of CpG island hypermethylation has been associated with increasing age in humans[44]. This may be contributory to the development of cancer as individuals age. A similar phenomenon of accumulated CpG island hypermethylation has not been demonstrated in dogs. In this series of lymphoma samples, there was no statistically significant association between age and the degree of hypermethylation. This phenomenon should be studied further in normal and diseased populations of dogs with sufficient sample size to answer this question.

Analysis of hypermethylation by site identified no relationship between Working Formulation classification or survival in these dogs. This is not surprising, as the hypermethylation had no effect on expression of canine *DLC1*. Hypermethylation has been shown to be a frequent finding in all of the forms of human NHL, with differences occurring primarily in degree of hypermethylation and gene sets affected [45-47]. The present cases were only classified by Working Formulation criteria after the fact. If comparative studies are to bear fruit on a mechanistic level, disciplined classification must become the norm for veterinary pathologists and clear prognostic significance must be attached to the different classifications. Development of diagnostic technology for flow-cytometry, antigen-receptor gene rearrangement, and, now, epigenetic alterations of cells will define the points of origin of canine NHL in B-cell biology that have already been defined in human NHL[5,48]. Only with this information will the comparative model be complete.

## Conclusion

The results of this study further characterize the similarities and differences between the human and canine *DLC1 *gene. The cDNA sequences of canine and human *DLC1 *are highly similar. The promoter regions of both human and canine *DLC1 *have important similarities in content of methylation sensitive transcription factors. Significantly, the density of Sp1 sites in dogs is more numerous and more concentrated at the 5' end of the promoter, potentially making the canine CpG island more resistant to hypermethylation. *DLC1 *is not silenced in canine NHL, but the statistically significant relationship with the malignant phenotype suggests that hypermethylation may be used as a biomarker for neoplasia in abnormal lymphoid populations. The clear association between hypermethylation and the malignant phenotype, taken with previously published data demonstrating global hypomethylation,[28]. demonstrate that methylation patterns are altered in canine forms of NHL in a similar manner to human NHL. Current research in the author's laboratory using high-throughput technology will begin to establish the patterns of hypermethylation associated with specific forms of lymphoma in dogs, and further define the comparative framework of NHL etiology, pathobiology, and therapy between dogs and humans.

## Methods

### *In Silico *methods

A region of the canine genome corresponding to the sequence surrounding the promoter region of the human *DLC1 *gene (NM_006094) was identified as previously described[29]. This region of DNA, located on chromosome 16 from 39,535,425 to 39,536,000 was considered to contain the promoter sequence for development of the subsequent studies.

A list of 7660 putative transcription factor motifs was obtained from the SigScan database of the Bioinformatics and Molecular Analysis Section of the Center for Information Technology, National Institutes of Health. http://www-bimas.cit.nih.gov/. Using the regular expressions pattern matching function in MATLAB^® ^version R2007a http://www.mathworks.com/, these motifs were compared with the canine and human *DLC1 *DNA sequences and the start and stop sites were recorded for all perfect matches. This resulted in 83 and 87 matched transcription factor motifs for the canine and human sequences, respectively. Of these, 10 canine and 14 human motifs represent consensus motifs that could potentially represent different specific transcription factor binding sites. There were 60 canine and 63 human motifs that were unique. The locations of CpG dinucleotides were determined in a similar fashion.

The sequence of canine *DLC1 *was translated using an online Open Reading Frame Finder http://www.ncbi.nlm.nih.gov/gorf/gorf.html. The sequences were compared to the human *DLC1 *sequence available in Gen-Bank using ClustalW http://www.ebi.ac.uk/clustalw/index.html, an online alignment program[49]. Protein functional group analysis was performed using an online analysis program http://www.ebi.ac.uk/Tools/InterProScan/[50].

### Patient samples

The case samples were collected from dogs presenting to the University of Missouri-Columbia Veterinary Medical Teaching Hospital meeting the ethical standards of, and in compliance with, IACUC requirements. Samples were obtained by lymphadenectomy or Tru-Cut needle biopsy. Samples were divided in two portions, placed in RNALater (Ambion, Inc., Austin, TX), and stored immediately at -80°C for later processing. The majority of the dogs in the study underwent complete staging, including CBC, plasma chemistry profile, bone marrow cytology, thoracic radiographs, abdominal ultrasound, and biopsy for definitive diagnosis and immunophenotyping. Staging was not required for inclusion. The normal lymphocytes used for this study were from laboratory dogs used for a concurrent protocol[51], blood samples of healthy volunteer dogs, or node samples harvested immediately after euthanasia of donated animals without lymphoma. Peripheral blood mononuclear cells were isolated over a ficoll-hypaque (Sigma-Aldrich, St. Louis, MO) gradient and preserved at -80°C until analysis. All available samples were reviewed by a single pathologist (LMB) to assign a Working Formulation diagnosis.

### DNA and RNA preparation

Sample collection and preparation: Samples were divided into two aliquots immediately after thawing in an ice bath. DNA and RNA were harvested using Qiagen DNeasy Tissue and RNeasy according to the provided protocols (Qiagen, Inc. USA. Valencia, CA). Briefly, for RNA the tissue was stabilized in RNAlater reagent to arrest RNase activity. Samples were homogenized and lysed, DNA sheared, and ethanol added. The solution was centrifuged in a proprietary spin column to bind the total RNA. The RNA was washed three times and, finally, eluted in RNase-free water. DNA was extracted using the Qiagen DNeasy Tissue kit (Qiagen, Inc. USA. Valencia, CA), and then bisulfite treated using the Zymo Research EZ DNA Methylation Gold kit (Zymo Research Corporation, Orange, CA). DNA from normal canine lymph nodes was treated with SssI and SAMe to methylate all CpG dinucleotides prior to bisulfite treatment in the sequence and serve as a positive control.

### Sequencing of cDNA

A spleen sample was removed from RNA later, ground in liquid Nitrogen using a precooled mortar and pestle and RNA was extracted using the RiboPure RNA extraction Kit (Ambion, Austin, TX, USA) following the manufacturer's specifications. The frozen powder was mixed immediately with 1 mL TRI buffer (to make 5% W/V homogenate) containing guanidinium thiocyanate. The homogenate was transferred into RNase free 1.5 microcentrifuge tubes (supplied in the kit) and quickly homogenized by vortexing at maximum speed. The homogenate was then mixed well by vortexing at maximum speed with 200 μL of chloroform solution, incubated for 5 minutes at room temperature, and centrifuged at 12,000 g for 30 seconds. The aqueous phase (containing the RNA) was recovered and mixed with 200 μL of 100% ethanol and applied to a glass fiber filter. The RNA attached to the spin column was washed twice with 500 μL of wash solution to remove residual contaminants. Purified RNA was eluted from the column in 100 μL Elution Buffer

Genomic DNA contaminant was eliminated by treatment with rDNase I using the DNA-free kit (Ambion, Austin, TX, USA). Total RNA (about 10 ug) was treated with rDNase I consisting of 1 μL (2 units) rDNase I and 5 μL of 10× DNase I buffer. The reaction mixture was incubated in a thermocycler at 37°C for 30 minutes then 5 μL of inactivation reagent was added to terminate the reaction and precipitate the rDNase. The mix was centrifuged at 10,000 g for 1.5 minutes and the RNA was transferred to a new RNase and DNase free microcentrifuge tube. The RNA was stored at -80°C until use.

The Ambion FirstChoice RLM-RACE (rapid amplification of cDNA ends) (Ambion, Inc. Austin Texas) kit was used to isolate the full length mRNA of the canine homolog of *DLC1 *and perform 5' and 3' RACE. Briefly, total RNA was treated with calf intestinal phosphatase to remove free 5' phosphates from rRNA, fragmented mRNA, tRNA, and DNA fragments. The 5' cap was removed with tobacco acid pyrophosphatase and a 45 base RNA adaptor was added to the complete mRNA. random primed reverse transcription reaction and nested PCR was used to amplify the remainder of the 5' end. A similar procedure was employed on the 3' end of the full length mRNA by placing a 3' adaptor and repeating the random-primed reverse transcription and nested PCR. Exonic primers that overlapped by a minimum of 50 bases were designed according to Ambion recommendation to amplify the regions of the mRNA between the 5' and 3' amplified segments. The PCR product was enzymatically purified with Exo-SapIt (Amersham, USA). PCR products were incubated for 30 minutes at 37°C and the reaction was terminated by incubation at 80°C for 15 minutes. (Amersham, USA). PCR product were further purified with Zymo Clean and Concentrator kit (Zymo Research Corporation, Orange, CA) and eluted in 10 μL of HyPure water. Clean PCR product was sequenced by cycle sequencing using big-dye terminator. The reaction was carried out in a 10 μL reaction containing 4 μL big-dye, 4 μL PCR, and 2 μL (3.2 pmol) primer. The mixture was cycled as follows: initial denaturation at 96°C for 2 minutes, and 36 cycles of 96°C for 10 seconds, 50°C for 10 seconds, and 60°C for 4 minutes.

### COBRA and MSP

The MethPrimer http://www.urogene.org/methprimer/index1.html website was used to locate a primer region at the 5' end of the CpG island to serve as the left primer[52]. Because the sequence contains a single CpG dinucleotide, a second primer was designed to be complementary to a TpG sequence at the same location. The right primer was constructed 187 bp 3' to the first primer pairs, and contains a single CpG dinucleotide as well, necessitating design of TpG complementary primer as well. The product size of these primers is 187 bp and contains one BstuI cut-site yielding fragments of 116 bp and 71 bp, one TaqaI cut site yielding fragments of 128 bp and 59 bp, and one HpyCh4IV cut site yielding fragments of 160 and 27 bp. The primers used for COBRA were (5' to 3'): forward, TATAGTTTTAGGCGGTGTTTTGG and TATAGTTTTAGGTGGTGTTTTGG and reverse, CCCAAAATCCAACTCAAAATTTACG and CCCAAAATCCAACTCAAAATTTACA. PCR was performed at an annealing temperature of 60°C for 60 s, an extension temperature of 72°C for 60 s, and a melting temperature of 95°C for 60 s, repeating for 36 cycles. The PCR product was purified with the Zymo Clean and Concentrator kit (Zymo Research Corporation, Orange, CA) and eluted in 20 μL of HyPure water. For BstuI, 10 μL of PCR product was added to 2.5 μL of Buffer 2, 1 μL of BstuI, and 11.5 μL of HyPure water, and incubated at 60°C for 4 h. For TaqaI, 8 μL of PCR product was added to 2.0 μL of Buffer 3, 1 μL of 10× bovine serum albumin, 1.5 μL of TaqaI, and 7.5 μL of HyPure water, and incubated at 65°C for 4 h. For HpyCh4IV, 8 μL of PCR product was added to 2.0 μL of Buffer 3, 1.5 μL of HpyCh4IV, and 8.5 μL of HyPure water, and incubated at 37°C for 4 h. Controls were bisulfite-treated DNA from normal lymphocytes (negative) and bisulfite-treated SssI-treated DNA from normal lymphocytes (positive). The PCR products were run on a 1.5% agarose gel with ethidium bromide for visualization. MSP was performed as previously described for this gene[29]. The primers used were: unmethylated forward TGGTTTTAAGTTTAGTGGTTAGTGG; unmethylated reverse CCTTATCAAACCAATACCTATCATT; methylated forward GCGGTTTTAAGTTTAGTGGTTAGC; methylated reverse CCTTATCAAACCGATACCTATCGT. Results were analyzed using a gel documentation and analysis system (Kodak, Rochester, NY). Proportion of methylation for COBRA samples was calculated as the intensity of the cut bands divided by the total intensity of all bands in the lane. For MSP, a ratio of the intensity of the methylated band to the unmethylated band was calculated.

### Bisulfite sequencing

The PCR product of 5' COBRA primer set from three cases and a normal dog was amplified, purified with the Qiagen Clean and Concentrator kit (Qiagen, Inc. USA. Valencia, CA) and eluted in 10 μL of HyPure water. Using the Invitrogen TA Cloning kit (Invitrogen Corporation, Carlsbad, CA), the product was transfected into competent cells provided for transformation[29]. Single colonies of transformed cells were selected and grown overnight in enrichment broth with ampicillin. Plasmid DNA was extracted using the Qiagen miniprep kit (Qiagen, Inc. USA. Valencia, CA), and the presence of an insert, indicating a transformed cell, detected by PCR. Five clones of each purified plasmid DNA were submitted with universal primers where they were sequenced by the DNA Core using a standard dideoxynucleotide sequencing technique. CpG dinucleotides within the sequences were mapped and labeled as methylated if cytosine remained unconverted, and unmethylated if a thymidine was in a cytosine position.

### Expression analysis

Primers were designed that reside in two adjacent exons, spanning an intron, near the 3' end of the mRNA. Reverse-transcriptase PCR was used to create a cDNA copy of the mRNA. This cDNA copy was amplified in tandem with GAPDH cDNA to yield relative copy numbers in SYBR-Green Real-Time PCR. Primer sequences were as follows: GAPDH forward GTGACTTCAACAGTGACACC; GAPDH reverse CCTTGGAGGCCATGTAGACC; DLC1 forward CTCACCTACATGTGCAGAGC; DLC1 reverse ACAACTTCAGCTGCACACAG. This was performed in five normal dog samples, as a control population, and all NHL samples. Assays were run in triplicate to avoid inter-assay variability. Data were evaluated using the ΔΔCt method.

### Statistical analysis

The relationship between degree of methylation and level of expression and degree of methylation and age were evaluated using a linear correlation model. Methylation density of each NHL sample was compared to expression of the mRNA relative to normal lymphoid tissue using a Mann-Whitney rank sum test. Association between methylation at each restriction enzyme or MSP site and the neoplastic phenotype or lymphoma subtype results was examined using the Fisher's Exact Test. Differences in survival were calculated for dogs receiving a CHOP chemotherapy protocol which did or did not have methylation at each restriction site and MSP analysis using the Kaplan Meier Log-Rank Analysis. Significance was set at P = 0.05.

## List of abbreviations

DLC1: Deleted in Liver Cancer 1; COBRA: combined bisulfite restriction analysis; MSP: methylation-specific PCR; NHL: non-Hodgkin's lymphoma; NSCLC: non-small cell lung carcinoma; RhoGAP: Rho-GTPase Activating Protein; SAM: sterile α motif; SRE: serum response element; START: steroidogenic acute regulatory-related lipid transfer; TSP2: thrombospondin 2.

## Authors' contributions

JNB was the principal investigator of this project and performed the primary methylation analysis, expression analysis, statistical analysis, and manuscript preparation. MJ performed the cDNA sequencing under the supervision of JNB. LMB performed the pathology slide review and classification. GLA performed the promoter analysis. KHT and FR provided critical protocol support and primer design necessary to complete the project. KCR performed case follow-up for outcome analysis. CJH, MRL, and CWC provided mentoring and critical input to project design, access to resources, manuscript editing, and intellectual contribution. WVR and JAV provided case management and sample collection for the project. All authors provided editorial contribution and approved this manuscript.
